# Unintended pregnancies and the use of maternal health services in southwestern Ethiopia

**DOI:** 10.1186/1472-698X-13-36

**Published:** 2013-09-08

**Authors:** Yohannes Dibaba Wado, Mesganaw Fantahun Afework, Michelle J Hindin

**Affiliations:** 1 Department of Population & Family Health, Jimma University, Jimma, Ethiopia; 2 School of Public Health, Addis Ababa University, Addis Ababa, Ethiopia; 3 Johns Hopkins Bloomberg School of Public Health, Baltimore, USA

**Keywords:** Unintended pregnancy, Antenatal care, Delivery care, Southwestern Ethiopia

## Abstract

**Background:**

The benefits of maternal health care to maternal and neonatal health outcomes have been well documented. Antenatal care attendance, institutional delivery and skilled attendance at delivery all help to improve maternal and neonatal health. However, use of maternal health services is still very low in developing countries with high maternal mortality including Ethiopia. This study examines the association of unintended Pregnancy with the use of maternal health services in Southwestern Ethiopia.

**Methods:**

Data for this study come from a survey conducted among 1370 women with a recent birth in a Health and Demographic Surveillance Site (HDSS) in southwestern Ethiopia. An interviewer administered questionnaire was used to gather data on maternal health care, pregnancy intention and other explanatory variables. Data were analyzed using STATA 11, and both bivariate and multivariate analyses were done. Multivariate logistic regression was used to assess the association of pregnancy intention with the use of antenatal and delivery care services. Unadjusted and adjusted odds ratio and their 95% confidence intervals are reported.

**Results:**

More than one third **(**35%) of women reported that their most recent pregnancy was unintended. With regards to maternal health care, only 42% of women made at least one antenatal care visit during pregnancy, while 17% had four or more visits. Institutional delivery was only 12%. Unintended pregnancy was significantly (OR: 0.75, 95% CI, 0.58-0.97) associated with use of antenatal care services and receiving adequate antenatal care (OR: 0.67, 95% CI, 0.46-0.96), even after adjusting for other socio-demographic factors. However, for delivery care, the association with pregnancy intention was attenuated after adjustment. Other factors associated with antenatal care and delivery care include women’s education, urban residence, wealth and distance from health facility.

**Conclusions:**

Women with unintended pregnancies were less likely to access or receive adequate antenatal care. Interventions are needed to reduce unintended pregnancy such as improving access to family planning information and services. Moreover, improving access to maternal health services and understanding women’s pregnancy intention at the time of first antenatal care visit is important to encourage women with unintended pregnancies to complete antenatal care.

## Background

The fifth Millennium Development Goal (MDG) calls for a reduction in maternal mortality by 75% between 1990 and 2015. According to the Maternal Mortality Estimation Inter-Agency Group (MMEIG), an estimated 287, 000 maternal deaths occurred in 2010 globally, down from 543,000 in 1990 [1]. The report showed that Sub-Saharan Africa (SSA) accounted for 56% of the deaths and had the highest Maternal Mortality Ratio (MMR) at 500 maternal deaths per 100, 000 live births. The pace of decline in SSA was also slower than the 5.5% annual pace of decline required to meeting the MDG goal on maternal mortality [1].

The benefits of maternal health care to maternal and neonatal health outcomes have been well documented. Attendance of antenatal care, institutional delivery and skilled attendance at delivery all help to improve maternal and neonatal health [2-4]. Antenatal care provides an opportunity to deliver interventions for improving maternal nutrition, providing health education, and encouraging skilled attendance at birth [5-7]. There is evidence that access to skilled assistance and well equipped health institutions during delivery can reduce maternal mortality and morbidity and improve pregnancy outcomes [8-11]. Delivery assisted by skilled provider is an important intervention in reducing maternal mortality, and one of the MDG indicators to track national effort towards safe motherhood. However, use of these interventions in most of the countries with high maternal and child mortality is still unacceptably low.

Ethiopia has one of the highest maternal mortality in the world and low utilization of maternal health services. According to the 2011 Ethiopian Demographic and Health Survey (EDHS), maternal mortality was 676 deaths per 100,000 live births, only 34% and 10% of women used antenatal care and delivery care respectively [12]. Although the Ethiopian Health Policy focuses on public health preventive and promotive interventions in the major areas of public health including maternal and child health, progress in maternal health is still very low. Maternal health care goals and targets were outlined in the Health Sector Development Program (HSDP) and National Reproductive Health Strategy, but achievements were short of the targets [13,14]. Since 2003, the government has been implementing a community health program called Health Extension Program (HEP) with the aim of providing preventive and promotive services such as maternal health services to the rural population. This program is appearing successful recently as improvements are seen in key health indicators such as child mortality and family planning [12].

Several studies have examined the effects of individual, household and community level factors on the use of maternal health services in developing countries [15-19]. However, very few have considered the effects of unintended pregnancy in the setting of sub-Saharan African countries. Unintended pregnancy is a pregnancy that was not wanted at the time conception occurred, irrespective of whether or not contraception was being used [20]. Available evidence shows that a significant level of unintended pregnancy exists in all developed and less developed countries. According to the Guttmacher Institute, about 80 million unintended pregnancies occur among women in developing countries [21]. Analysis of DHS data also showed that the magnitude of unintended pregnancies in developing countries ranged from 14% to 62% of all births [22]. The highest rate of unintended pregnancy occurs in Sub-Saharan Africa, where about 86 unintended pregnancies occur for every 1000 women of reproductive age [23]. In Ethiopia, according to the 2011 EDHS, 25% of women with births in the five years before the survey and 32% of current pregnancies were reported as unintended [12].

Unintended pregnancy is an important public health problem that predisposes women to maternal deaths and illnesses mainly through unsafe abortions and poor maternity care. With regards to maternal health, several studies have linked unintended pregnancy with adverse maternal behavior during pregnancy including delayed and inadequate antenatal care use [24-27], maternal depression and anxiety [28-30], and smoking and drinking behaviors during pregnancy [31,32]. There is a large body of research that documents the negative consequences of unintended pregnancy on use of antenatal care services in developed countries [27,32,33]. Most of these studies showed that unintended pregnancy is associated with late initiation and inadequate use of antenatal care services.

However, there are few studies from developing countries on the subject and many of the existing ones are based on DHS data [24,25,34-37]. Some of these studies found that women with unintended pregnancies are less likely to use antenatal care services and or less likely to receive adequate antenatal care [24,25,35]. However, an inconsistent pattern of relationships between unintended pregnancy and antenatal care utilization was reported in some others [36,37]. For instance, in a multi-country study using DHS data from five developing countries, Marston and Cleland found that women experiencing unintended pregnancies were significantly more likely to delay antenatal care in three of the five countries but not in two others (Egypt and Bolivia). This study also assessed the relationship between unintended pregnancy and delivery care utilization, finding increased odds of unsupervised delivery for unwanted children in Peru and Kenya, but not in the other 3 countries [36]. The few studies that assessed the relationship between unintended pregnancy and delivery care utilization found no or marginal association between pregnancy intention and delivery care utilization [36-38]. Overall, existing studies on the subject are either limited or have reported inconclusive findings. It is thus important to assess how unintended pregnancy influences maternal health care in situations of high unintended pregnancy and low maternal health care. In this study, we examine the associations of pregnancy intention with the use of maternal health services in Southwestern Ethiopia.

## Methods

This is a cross-sectional study conducted in Gilgel Gibe Health and Demographic Surveillance System (HDSS) in Jimma zone, Southwestern Ethiopia, which is located at 260 kilometers to the southwest of Addis Ababa. The study population was women, of age 15–49 years, with a live birth in the two years before the survey (March 2012). The HDSS at the Gilgel Gibe site in southwestern Ethiopia is used to collect vital events data by Jimma University. Accordingly, data on all births occurring in the site is collected through an update of multiple times in a year. Participants were then drawn from eleven sub-districts (kebeles^a^) in HDSS using a simple random sampling procedure. In this HDSS area consisting of over 55,000 people, there were 3293 women with a live birth in the 2 years before the survey date, of which 1456 were randomly selected for the present study. A sample size of 1456 was calculated for the study using two population proportion formula, assumptions of the prevalence of ANC use (50%) and difference of 8% between women with intended and unintended pregnancies, and power of 80%.

Data were collected by ten trained female data collectors who had a diploma level training and experience in data collection. They were closely supervised by supervisors who had better experience in data collection. The data collectors had five days of training on how to administer the questionnaire including practice interviewing, role playing and addressing ethical issues. After the training, a pilot study was done and information from the pilot study was used to finalize the questionnaire.

A structured questionnaire originally developed in English and translated to local language (Oromo) was used to collect data. Data on maternal health care, pregnancy intention and other explanatory variables were gathered using interviewer administered structured questionnaire. Ethical approval was obtained from the College of Health Sciences, Addis Ababa University. Moreover, participants were asked for informed consent, and participation in the study was fully voluntary. Consent form was translated to local language (Oromo) and was read to every participant before starting the interview.

In this study, maternal health care refers to the use of antenatal care during pregnancy and delivery at a health facility. Women were asked whether they have used any antenatal care during their most recent pregnancy and whether they delivered at a health facility. The two variables were measured on a binary scale as ‘yes’ for those who used the services, and ‘no’ for those who did not use the services. For antenatal care, we examined two measures of women’s use of antenatal care. The first is receiving any antenatal care, named hereafter as ‘antenatal care use’. The second is making adequate number of antenatal care visits – defined based on recommendations from a World Health Organization (WHO) which stated that a woman without complications should have at least four antenatal care visits, the first of which should take place during the first trimester.

The key independent variables were measured in the following ways. Pregnancy intention was measured using the standard DHS approach, which asks women to recall their feelings at the time they became pregnant; “At the time you became pregnant, did you want to become pregnant then, did you want to wait until later, or did you not want to have any (more) children at all?”. The responses are: (1) wanted then “intended”, (2) wanted to happen later “mistimed”; (3) did not want at all “unwanted”. Mistimed and unwanted pregnancies were then grouped together as “unintended pregnancies”. Other explanatory variables included age (coded as 15–24, 25–34 and 35–49), women’s education (coded as no education, primary and secondary and above), place of residence, wealth index, distance from health facility, presence of pregnancy related morbidity, parity, ever use of modern family planning and time of pregnancy recognition. The wealth index was computed from ownership of the following household assets: radio, television, electricity, refrigerator, toilet, farm land, and quantity of animals such as cattle, sheep, and goats. Principal Component Analysis (PCA) was run, and four principal components with eigenvalues greater than one were summed to obtain wealth index values [39]. The resulting index was then divided into three categories representing poor, middle and wealthy. Distance from health facility was asked in walking hours or minutes; ‘How long it takes to walk on foot from their home to the nearest health facility providing maternal health services?’. Moreover, women were asked whether they have experienced any illness during pregnancy. Time of pregnancy recognition refers to the approximate gestational age at which the women found out that she was pregnant.

Similarly, women’s participation in decision making was measured by asking the following questions; “who makes decisions in your household about: (1) obtaining health care for yourself; (2) large household purchases; (3) household purchases for daily needs; and (4) visits to family or relatives?”. The responses were: (1) respondent alone, (2) respondent and husband/partner, (3) husband/partner alone, (4) someone else. Women are considered to participate in a decision if they usually make that decision alone or jointly with their husbands. Then a composite index was constructed by grouping women into two categories: women who have any say (alone or jointly) in all four household decisions, indicating a higher level of empowerment, and women who do not have any say in one or more decisions.

### Data analysis

Data were analyzed using STATA software version 11. First, a descriptive analysis of the characteristics of study population was made. Bivariate analysis was done to compare use of maternal health care among different groups using chi-square test. Multivariate logistic regression was done to identify factors that are independently and significantly associated with use of antenatal care and delivery care services. Unadjusted and adjusted odds ratio and their 95% confidence intervals are reported. Multicollinearity of variables was checked using variance inflation factor.

## Results

### Socio-demographic characteristics of the respondents

Of 1456 eligible women, 1370 were successfully interviewed with a response rate of 94%. The study participants had a median age of 27 years. A majority of them were rural (74%), married (98%), Muslim (92%), and had no formal education (75%). The average number of children ever born was 4.3(±2.3), and nearly 44% had given birth to 5 or more children. About 65% of women reported that their last pregnancy was intended, while for 25% and 10% of women their last pregnancy was mistimed and unwanted (unintended) respectively. A higher proportion of women of age 35–49, rural women, women with no education, women with high parity, and women living farther from health facility reported unintended (mistimed and unwanted) pregnancy (Table 1).

**Table 1:** Percent distribution of women by pregnancy intention according to selected background variables (n = 1370)

**Socio-demographic variables**	**N**	**Intended pregnancy**	**Mistimed pregnancy**	**Unwanted pregnancy**
Age				
15–24	373	71.9	24.1	4.0
25–34	790	62.6	27.0	10.4
35+	207	61.8	18.8	19.3
Educational status				
No formal education	1021	62.0	26.5	11.5
Primary	305	71.8	22.0	6.2
Secondary & above	44	88.6	9.1	2.3
Residence				
Rural	1012	61.8	26.8	11.4
Urban	358	74.0	19.8	6.2
Marital status				
Currently married	1342	65.1	25.1	9.8
Widowed or divorced	28	64.3	17.8	17.9
Participation in decisions				
No	643	61.2	28.5	10.3
Yes	727	68.4	21.9	9.8
Parity				
1-2	358	80.6	17.2	2.2
3-4	410	65.1	27.3	7.6
5^+^	602	52.4	29.1	18.5
Distance from health facility				
< 1 hour	744	69.9	20.7	9.4
> 1 hour	626	60.1	29.2	10.7

### Use of maternal health services

Table 2 shows the percentages of women who used antenatal care and delivery care services by different background variables. Forty two percent of women used antenatal care for their most recent pregnancy. The table shows that antenatal care use is higher among younger women age 15–24 (45%), urban women (62%), women with secondary and above education (82%), women in the highest wealth tertile (59%), women who intended the pregnancy (45%), women participating in all household decisions (46%), women with first parity (50%), and women living nearer to health facility (46%). With regards to delivery care, 12% of women delivered their last child in a health facility; 2% in a hospital, 9% in a health center, and 1% in a private clinic. Twelve percent of women were assisted by a skilled provider - a doctor, a nurse or a midwife. Health facility delivery was higher among younger women age 15–24 (16%), urban women (25%), women with secondary and above education (48%), women in the highest wealth tertile (23%), women who intended the pregnancy (14%), women participating in all household decisions (14%), women with lower parity (19%), and women living nearer to health facility (19%) (Table 2).

**Table 2:** Percentage distribution of women by antenatal care and delivery care use

**Variables**	**Antenatal care (%)**	**P**	**Delivery care (%)**	**P**	**Number of women**
Age					
15–24	44.8	0.05	16.1	0.02	373
25–34	42.2		11.0		790
35+	34.3		9.7		207
Educational status					
No education	36.0	0.001	9.0	0.001	1021
Primary	54.8		17.7		305
Secondary & above	81.8		47.7		44
Residence					
Rural	34.6	0.001	7.6	0.001	1012
Urban	61.7		25.1		358
Pregnancy intention					
Intended	45.3	0.001	14.1	0.005	892
unintended	34.9		8.6		478
Wealth index					
Low	25.0	0.001	4.1	0.001	456
Middle	41.4		10.1		457
Upper	59.0		22.6		457
Participation in decisions					
No	36.4	0.001	10.7	0.12	643
Yes	46.4		13.5		727
Parity					
1-2	48.0	0.001	19.0	0.001	315
3-4	44.6		11.0		410
5^+^	35.9		9.0		602
Distance from health facility					
< 1 hour	53.9	0.001	19.3	0.001	685
> 1 hour	29.5		5.1		685
Illness during pregnancy					
No illness	40.0	0.04	15.0	0.05	976
Illness	46.0		11.1		394
Total	41.7		12.2		**1370**

### Multivariate associations of unintended pregnancy and maternal health care

In the logistic regression analysis pregnancy intention was associated with use of antenatal care, but not with delivery care, after adjusting for socio-demographic variables. Considering antenatal care use, women with unintended pregnancy were 25% less likely (OR: 0.75, 95% CI, 0.58-0.97) to have used antenatal care as compared to women with intended pregnancy. Unintended pregnancy was associated with delivery care at the bivariate level, but not in multivariate analysis. Factors other than pregnancy intention significantly associated with both antenatal care and delivery care include; women’s education, wealth tertile, residence, ever use of modern family planning and distance from health facility. Women’s participation in decision-making was associated with antenatal care but not with delivery care. Women who have a say in all four decisions were 34% more likely to have used antenatal care as compared to women who did not have a say in all household decisions. For delivery care, use of antenatal care was also significantly associated (Table 3).

**Table 3:** **Adjusted odds ratios of Antenatal** &**Delivery care use by pregnancy intention and other characteristics, SW Ethiopia 2012 (n = 1370)**

**Variables**	**Antenatal care**^**1**^	**Delivery care**^**1**^
	**OR (95% CI)**	**OR (95% CI)**
Pregnancy intention		
Intended	Ref	Ref
unintended	0.75 (0.58–0.97)*	0.71 (0.47–1.08)
Age		
15–24	Ref	Ref
25–34	1. 03 (0.72–1.47)	0.78 (0.47–1.29)
35+	0.79 (0.49–1.29)	0.81 (0.39–1.69)
Educational status		
No education	Ref	Ref
Primary	1.45 (1.07–1.96)*	1.05 (0.70–1.59)
Secondary & above	3.04 (1.33–6.97)**	2.31(1.13–4.75)*
Residence		
Rural	Ref	Ref
Urban	1.56 (1.14–2.14)**	1.68 (1.11–2.55)**
Parity		
1–2	Ref	Ref
3–4	1.07 (0.73–1.56)	0.66 (0.38–1.11)
5^+^	0. 96 (0.63–1.45)	0.79 (0.44–1.43)
Wealth index		
Low	Ref	Ref
Middle	1.64 (1.21–2.21)**	1.52 (0.84–2.74)
Upper	2.31 (1.64–3.26)***	2.40 (1.32–4.36)**
Participation in decisions		
No	Ref	Ref
Yes	1.34 (1.06–1.70)*	1.05 (0.73–1.51)
Distance from health facility		
< 1 hour	Ref	Ref
> 1 hour	0.66 (0.50–0.87)**	0.55 (0.34–0.89)*
Pregnancy related morbidity		
No	1	1
Yes	1.32 (1.02-1.72)*	1.40 (0.96–2.05)
Ever use of family planning		
No	1	1
Yes	2.58 (1.95–3.40)***	2.27 (1.56–3.31)***
Antenatal care use		Ref
No	-	2.41 (1.63–3.58)***
Yes		

*significant at P < 0.05 **P < 0.01 ***significant at p < 0.001.

^1^Adjusted for age, education, residence, parity, wealth, and participation in decisions.

The median duration of pregnancy at the time of first antenatal care visit was 5 months. Only 17% of women made four or more visits and 13% started ANC visit in the first trimester (results not shown). In the multinomial logistic regression analysis, we compared women who had adequate antenatal care (4 or more ANC visits), women who made inadequate visits (1–3 ANC visits) with women who did not make any ANC visits. The results show that pregnancy intention is significantly related to receiving adequate antenatal care. Women with unintended pregnancies were 33% less likely (OR: 0.67, 95% CI, 0.46-0.96) to receive adequate antenatal care as compared to women who intended their pregnancy, after adjusting for socio-demographic variables.

Other variables significantly associated with use of adequate antenatal care include; women’s education, wealth, distance from health facility, and the time of pregnancy recognition. Women with primary education were 61% more likely (RR: 1.61, 95% CI, 1.09-2.38) to use adequate ANC, while women with a secondary and above education were nearly 4 times (RR: 3.96, 95% CI, 1.58-9.94) more likely to receive adequate ANC as compared with women with no education. Women from the richest wealth tertile were 1.82 times more likely to receive adequate antenatal care as compared to women from the lowest wealth tertile. Women living more than an hour travel distance from a health facility (health center or Hospital) were 41% less likely (RR: 0.59, 95% CI, 0.40-0.86) to receive adequate antenatal care. Similarly, women who recognized their pregnancy after 12 weeks were 39% less likely (RR: 0.61, 0.44-0.85) to receive adequate antenatal care (Table 4).

**Table 4:** Multinomial logistic regression of factors associated with receiving adequate vs inadequate antenatal care (RC = Non use of antenatal care)

**Variables**	**Less than four ANC visits**	**Four or more ANC visits**
	**RR (95% CI)**^**1**^	**RR (95% CI)**^**1**^
Unintended Pregnancy	0.86 (0.64–1.15)	0.67 (.46–.96)*
Age		
15–24	1.00	1.00
25–34	1.07 (.73–1.58)	1.07 (0.67–1.72)
35+	0.84 (.49–1.45)	0.94 (0.49–1.82)
Educational status		
No education	1.00	1.00
Primary	1.32 (0.95–1.87)	1.61 (1.09–2.38)*
Secondary & above	2.31 (0.92–5.76)	3.96 (1.58–9.94)*
Wealth index		
Low	1.00	1.00
Middle	1.64 (1.15–2.32)**	1.57 (1.02–2.43)
Upper	2.62 (1.76–3.88)***	1.82 (1.12–2.94)*
Urban residence	1.07 (0.73–1.55)	1.37 (0.90–2.10)
Parity		
1–2	1.00	1.00
3–4	0.99 (0.65–1.53)	1.17 (0.71–1.92)
5^+^	0.92 (0.58–1.46)	1.0 (0.58–1.74)
Participated in decisions	1.39 (1.06–1.82)*	1.27 (0.92–1.75)
> 1 hour walking distance from health facility	0.70 (0.51–0.96)**	0.59 (0.40–0.86)**
Recognized pregnancy after 3 months	0.89 (.68,1.17)	0.61 (0.44–0.85)**
Ever used family planning	2.19 (1.60–3.01)***	3.26 (2.30–4.62)***

*significant at P < 0.05 **P < 0.01 ***significant at p < 0.001.

^1^Adjusted for age, education, residence, parity, wealth, and participation in decisions.

## Discussion

This study examined the association between pregnancy intention and use of maternal health services in Southwestern Ethiopia. More than one third of women (35%) reported that their most recent pregnancy was not intended. Our results show that the level of maternal health care use is very low in the study area. Forty two percent of women used ANC, 12% delivered in a health facility and 12% had skilled assistance at delivery. This result is slightly higher than the findings of the 2011 EDHS which reported ANC use of 34% and institutional delivery of 10% [12]. In both cases, the level of maternal health care is very low in Ethiopia even compared with other sub-Saharan African countries. Moreover, very few women made four antenatal care visits and started antenatal care earlier in pregnancy. Considering the WHO recommendations of four focused antenatal care visits and an initial visit occurring before the fourth month of pregnancy, only 17% and 13% of women received antenatal care according to these recommendations respectively.

Our multivariate analysis showed that pregnancy intention is associated with use of antenatal care, but not with delivery care. Women with unintended pregnancies were 25% less likely to have used antenatal care as compared to women with intended pregnancy. Similarly, women who reported unintended pregnancies were 33% less likely to have had four or more antenatal care visits. Several previous studies from developed and developing countries found similar results regarding antenatal care [19-22]. However, there were also studies in which no or inconsistent associations were reported [31,32]. The reasons why women with unintended pregnancies do not use antenatal care or receive inadequate care is less clear. One hypothesis is that women with unintended pregnancies, compared to those with intended pregnancies, are likely to be less prepared emotionally and financially for the demands of pregnancy and childbearing and more likely to less care of themselves and the developing fetus during pregnancy [19]. Another line of argument is that women with unintended pregnancies recognize the pregnancy later [21]. It is also observed in this study that women with unintended pregnancies recognized the pregnancy on average a month later than women with intended pregnancies. Moreover, women with unintended pregnancies are more likely to be from rural areas where access to maternal health services is low. There could be socio-cultural influences such as fear of stigma given that a significant proportion of pregnancies to older and high parity women are unintended.

The association of unintended pregnancy with delivery care was attenuated once we controlled for other socio-demographic factors. We tested for multicollinearity using variance inflation factor (VIF), but did not see multicollinearity problem in the model although the VIF for parity increased as parity increased. Accordingly, a disaggregated analysis with parity showed that the association between unintended pregnancies with place of delivery is lower for high parity women, although it remained non-significant (result not shown). We also looked at whether disaggregating the two categories of unintended pregnancy (mistimed and unwanted pregnancy) in the multivariate analysis influences the association, and found that women with unwanted pregnancy (not mistimed pregnancy) were significantly less likely to go for delivery to health facilities than women with intended pregnancy. Overall, the association between pregnancy intention and delivery care use has been inconsistent in several previous studies [30-32].

Other factors independently associated with antenatal care and delivery care include women’s education, urban residence, wealth, distance from the nearest health facility and previous use of modern family planning services. In several studies from developing countries, such variables as women’s education, wealth and accessibility to health services were found to be factors that influence use of maternal health care [12-16]. Women’s education in particular has been consistently associated with use of maternal health services in several studies. The association of modern family planning use with both antenatal care and delivery care, and that of antenatal care with delivery care may indicate that women who have used maternal health service may be informed and connected to other maternal health services. Urban residence and distance to health facilities were significantly associated with use of antenatal care and delivery care in this study showing that accessibility to health services remains an important factor for maternal health care. Women’s participation in decision making is also associated with antenatal care use, but not with delivery care. Several studies have reported a lack of association between women’s autonomy and place of delivery care [40,41]. In a study from Kenya, Fotoso and colleagues reported that women’s participation in decision making was not associated with institutional delivery. A study from Guatemala [40] and Nepal [41] also reported that women’s participation in domestic decision making was not associated with use of delivery care.

Our study adds to the existing body of literature regarding the influences of unintended pregnancy on maternal health care in developing countries that will have important implications for maternal health. The study findings could be generalizable to similar settings in Ethiopia and sub-Saharan Africa. We recommend that future researches on the subject consider the two categories of unintended pregnancy (mistimed and unwanted) separately because differences exist between the two unintended pregnancy groups. The current study has some limitations. Most importantly, since it is a cross-sectional study, there is a possibility of under reporting and post-hoc rationalization in responding to pregnancy intention questions. There could also be recall bias in responding to questions on maternal care use, particularly on the timing and number of antenatal care visits.

## Conclusions

Our study found that unintended pregnancies influence use of antenatal care; women with unintended pregnancies were less likely to use ANC and less likely to receive adequate antenatal care. This indicates that pregnancy intention impacts maternal health behavior, as antenatal care is an important gate way to a continuum of maternal and child health care. Overall, the fact that more than one-third of women (35%) had unintended pregnancies and that use of maternal health services is low in the study area is a cause of concern. Efforts should be made to increase access to family planning information and services in order to reduce the high level of unintended pregnancies. This is especially true for women in the postpartum period because most of the unintended pregnancies reported in this study are mistimed pregnancies. Moreover, encouraging use of maternal health services and understanding women’s pregnancy intention at the time of first antenatal care visit is important to encourage women with unintended pregnancies to complete antenatal care and deliver in a health facility.

## Endnote

^a^Kebele is the smallest administrative unit in Ethiopia.

## Competing interests

The authors declare that they have no competing interests.

## Authors’ contributions

YDW, designed the study, monitored the data collection, analyzed the data, and wrote the first draft of the manuscript. MFA and MJH participated in the design of the study, supervised the whole process and reviewed and modified the drafts of the manuscript. All authors read and approved the final manuscript.

## Pre-publication history

The pre-publication history for this paper can be accessed here:

http://www.biomedcentral.com/1472-698X/13/36/prepub
